# The impact of using genetically modified (GM) corn/maize in Vietnam: Results of the first farm-level survey

**DOI:** 10.1080/21645698.2020.1816800

**Published:** 2020-09-30

**Authors:** Graham Brookes, Tran Xuan Dinh

**Affiliations:** a Agricultural Economist with PG Economics Ltd, Dorchester, UK; b Former Deputy Director General Crop Production Department, Ministry of Agriculture and Rural Development (CPD MARD), Vietnam

**Keywords:** Asian Corn Borer, insect resistant, herbicide tolerance, weed control, hand weeding, environmental impact quotient, active ingredient, costs, yield

## Abstract

This study assessed the farm-level economic and environmental impacts from the use of genetically modified (GM) corn in Vietnam (resistant to Lepidopteran pests of corn and tolerant to the herbicide glyphosate). It was largely based on a farmer survey conducted in 2018–19. The GM varieties out-performed conventional varieties in terms of yield by +30.4% (+15.2% if the yield comparison is with only the nearest performing equivalent conventional varieties) and reduced the cost of production by between US $26.47 per ha and US $31.30 per ha. For every extra US $1 spent on GM seed relative to conventional seed, farmers gained between an additional US $6.84 and US $12.55 in extra income. The GM maize technology also reduced insecticide and herbicide use. The average amount of herbicide active ingredient applied to the GM crop area was 26% lower (1.66 kg per ha) than the average value for the conventional corn area (2.26 kg/ai per ha) and in terms of the associated environmental impact of the herbicide use, as measured by the Environmental Impact Quotient (EIQ) indicator, it was lower by 36% than the average value applicable to the conventional corn area. Insecticides were used on a significantly lower GM crop area and, when used, in smaller amounts. The average amount of insecticide applied to the GM corn crop was significantly lower by 78% (0.08 kg/ai per ha) than the average value for the conventional corn area (0.36 kg/ai per ha) and in terms of the associated environmental impact of the insecticide use, as measured by the EIQ indicator, it was also lower by 77% than the average value for conventional corn (14.06 per ha).

## Introduction

Corn/maize crops that have been genetically modified (GM) to be tolerant to herbicides and resistant to some of the main corn pests became available to farmers in Vietnam in 2015, and in 2019, 92,000 ha (about 10.2% of the total crop) were planted to seed containing GM traits (statistical source: Crop Protection Department of the Ministry of Agriculture and Rural Development: CPD MARD).

This GM corn seed technology is tolerant to the herbicide glyphosate and offers resistance against the main (lepidopteran) corn pest; the Asian Corn Borer (ACB) – *Ostrinia furnacalis* but is also resistant to other lepidopteran pests such as corn earworms – *Helicoverpa zea*, common cutworms – *Spodoptera litura* and the Fall Armyworm (FAW) – *Spodoptera frugiperda*. The technology comprises two main trait alternatives. These are the combination of MON 89034 event, which contains two Bt proteins – Cry1A.105 + Cry2Ab2 for the control of lepidopteran pests coupled with the event NK603 (herbicide tolerance trait). The other trait combination is the Bt11 event which contains one Bt protein – Cry1Ab for lepidopteran pest control coupled with the GA21 event (herbicide tolerance trait). Both of these combinations of traits are referred to as “stacked” traits (herbicide tolerant (HT) and insect resistant (IR)). In relation to the IR events, MON 89034 is also sometimes referred to as “pyramidal” because it contains two novel Bt proteins, while the Bt11 event, which contains only one Bt protein is not referred to as “pyramidal.”

The “stacked-traited” seed technology is available to farmers in a limited number of yellow corn hybrid varieties that have been approved for use in Vietnam, notably NK66 BT/GT, NK 67 BT/GT, NK4300 BT/GT, NK7328 BT/GT, DK9955S, DK6919S, DK8868S, DK6818S, and CP 501S. The technology is, however, not available in some of the latest developed hybrid varieties, is not available in specialty varieties (e.g., waxy corn used in the starch manufacturing sector), in sweetcorn or in open pollinated varieties. In order to minimize the incidence of pests (especially those such as Ostrinia furnacalis which are polyphagous and feed on more than one crop species) becoming resistance to the IR technology, Insect Resistance Management (IRM) strategies are adopted. These are implemented through the technology provider. An appropriate IRM strategy includes the use of, what are referred to as, unstructured or structured refuges. An unstructured refuge is where other forms of non IR plants or wild hosts of the targeted pests are planted, whilst a structured refuge generally refers to a refuge planted as a separate block or field from the IR protected crop. Structured refuges can, for example, be provided by supplying a small package of refuge seed, along with the larger amount of IR-traited seed or a seed blend of the two types of seed. Along with a refuge-based IRM, a pyramid of more than one than one Bt protein can play a role in the IRM strategy.

This paper presents the findings of an ex-post analysis of the economic and environmental impacts (related to changes in pesticide use) that have arisen from the commercial adoption of this GM yellow corn hybrid seed in Vietnam. It represents the first analysis of the farm impact of using this corn seed technology relative to conventional corn pest and weed control practices and therefore provides a first opportunity to compare the impacts in Vietnam with evidence from other GM corn user countries. There is now a considerable body of evidence, much of it in peer-reviewed literature that quantifies broadly positive economic and environmental impacts associated with the adoption of GM crops, for example, as summarized in Klumper and Qaim,^1^ Finger et al,^2^ Brookes and Barfoot.^3^ Drawing for example on Brookes and Barfoot^3^ “*GM insect resistant (IR) traits have mostly delivered higher farm incomes through improved yields and many farmers have also had lower costs of production (especially less expenditure on insecticides). The GM herbicide tolerant (HT) technology has mostly contributed to higher levels of farm income by reducing costs of production, notably on weed control. However, in relation to HT crops, over reliance on the use of glyphosate and the lack of crop and herbicide rotation by some farmers, in some regions (notably North and South America), has contributed to the development of weed resistance. To address this problem, farmers have increasingly adopted more integrated weed management strategies incorporating a mix of herbicides, other HT crops and cultural weed control measures (eg, using other herbicides with glyphosate rather than solely relying on glyphosate, using HT crops which are tolerant to other herbicides, such as glufosinate and using cultural practices such as mulching”)*. A brief comparison of the findings from this Vietnamese study with the findings of similar research is presented in the latter discussion section.

### Baseline: Nature of Production, Pests and Conventional Control

**Figure 1.**
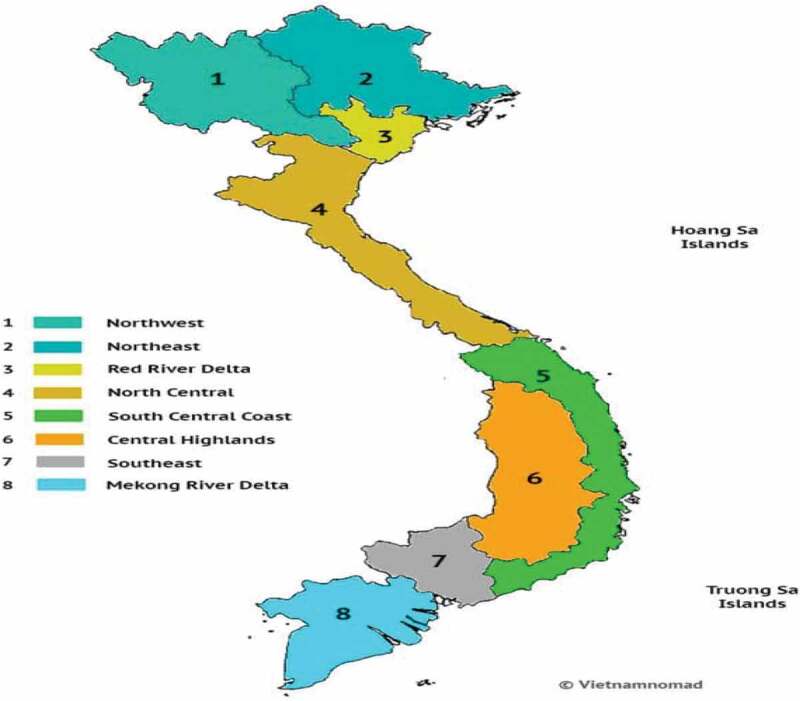
Map of main regions of Vietnam

The total corn crop is grown over two or three seasons per calendar year. The winter-spring season involves sowing of crops in the period late December to mid-February and harvesting March–May. The summer season has sowing in May–June and harvesting late August to early October and the autumn/winter season has sowing in late August–October and harvesting November to January. Corn is grown in all of the main regions of Vietnam (Fig. 1). Three seasonal crops are grown by many farmers in the North Mid-lands/Mountain region, North Central, Highlands, and South-East regions, with two seasonal crops commonplace in the Red River, Mekong Delta, and South-Central regions. In addition, in the main growing regions in the South (Central Highlands, South East, and Mekong Delta), some farmers also grow only one crop per year (planting in mid-May, harvesting in September). Overall, in the Red River, North Central and Central Coastal and North Midland/Mountain regions, approximately half of the crop is a spring crop, with about 30% autumn crop and 20% winter crop. The Autumn-Winter cropping season often follows the harvesting of rice or beans.

Almost all seed (more than 99.5%) used by yellow corn farmers is hybrid seed. Some open pollinated varieties are also sold, mainly in the waxy corn and sweetcorn parts of the market (source; personal communications with corn seed company representatives)

The corn crop is a mix of dryland (rain-fed) and irrigated, with most regions relying on rain-fed production – the main users of irrigation are in the Red River and Mekong Delta regions. The most common form of production is continuous corn over the two-three seasons, although some farms may rotate/switch out of corn in favor of competitor crops like soybeans, sweet potatoes, and vegetables. If corn is not grown continuously, the main crop grown in rotation is rice.

In terms of the structure of production, the average size of farm-growing corn is about half to 1 ha. The average area planted to corn is also half to one ha, with farmers usually planting a large majority of their total farm area to one crop per season (statistical source: CPD MARD). Corn is an important cash and staple crop for many rural households who typically grow it in conjunction with rice (their main staple crop).

About two-thirds of the conventional crop use herbicides for weed control with hand weeding used on the remaining third of the crop (source; Brookes^4^). A mix of both forms of weed control occurs on many farms. Hand weeding is mostly found in the North-Central and South-Central regions (areas of relatively lower weed pressure).

The main pest of corn in Vietnam is the ACB, which regularly causes economic levels of yield loss in 60% to 70% of the crop (statistical source: CPD MARD). As a result, this proportion of the crop regularly uses insecticides to control this pest and despite the application of insecticides, average yield losses are estimated (source: CPD MARD) in the range of −5% to −7%. The highest yield losses are found in the North Midland and Mountain, South-Central Coastal, and Mekong Delta regions. The Corn Earworm pest also causes yield losses in 10% to 12% of the crop (which can be up to −10% yield loss and mainly affects farms in the Mekong Delta, Red River Delta, and North Mid-land and Mountain regions; source; personal communications from seed industry representatives). The FAW pest is a relatively new pest to Vietnam and, in 2019, was reported to be affecting between 35% and 75% of the corn area in most regions (source: personal communications from seed industry representatives), with the highest levels of incidence in the regions of North Mid-lands/Mountainous, Red River, and North Central.

## Materials and Methods

The primary source of information has come from a survey of corn growers in Vietnam. Personal interviews with a sample of farmers in all corn-growing regions of the country were conducted in 2018–2019 by staff from CPD MARD and technical staff in the Provincial Departments of Crops and Plant Protection. In addition, the baseline section following this section draws on information from Brookes.^4^

The survey aimed to be reasonably representative of corn production by region, with a focus on regions where varieties containing GM traits were widely grown (Table 1). As a result, there was a deliberate bias introduced into the survey to reflect the distribution of plantings of GM corn and the sample. The target number of interviews for each region reflected the regional distribution of GM corn plantings in 2018 and as such resulted in a higher concentration of interviews undertaken in the regions of the Mekong Delta, Red River Delta, and South East relative to their respective regional importance to national corn production.

**Table 1.** Interview sample

Region	% of crop area	Target number of interviews	Number of interviews	% of interviews
Mekong Delta	3	140	140	19
Red River Delta	8	140	138	19
North Mid-lands/Mountains	44	210	199	27
North-Central	11	35	88	12
South-Central	7	35	30	4
Highlands	20	70	50	7
South-East	7	70	90	12
**Total**	**100**	**700**	**735**	**100**

Note: A total of 740 interviews were undertaken but five questionnaires were discarded due to inconsistent and/or incorrectly recorded data

Within each region, the aim was to divide the interviews equally between farmers growing GM corn and farmers planting conventional corn. The final outturn was a total of 395 farms using GM seed were interviewed, of which 56 of these farmers planted both GM and conventional corn seed varieties. The balance of 340 farmers interviewed planted only conventional corn.

The interviews were conducted mostly in the second half of 2018 and the first half of 2019, with completion (of approximately 60 interviews in the North Mid-lands and Mountainous region) by the end of October 2019.

The methodology used for assessing the environmental impact associated with pesticide use changes with GM corn in Vietnam examines changes in the volume (quantity) of pesticide applied and the use of the Environmental Impact Quotient (EIQ) indicator (Kovach et al^5^ and annually updated). The EIQ indicator provides an improved assessment of the impact of GM crops on the environment when compared to only examining changes in volume of active ingredient applied, because it draws on some of the key toxicity and environmental exposure data related to individual products, as applicable to impacts on farm workers, consumers and ecology. Drawing on Brookes and Barfoot^3 “^*The authors acknowledge that the EIQ is only a hazard indicator and has important weaknesses (see for example, Peterson and Schleier^6^ and Kniss and Coburn.^7^). Nevertheless, since assessing the full environmental impact of pesticide use changes with different production systems is complex and requires substantial collection of (site-specific) data (eg, on ground water levels, soil structure), it is not surprising that no such depth of data is available to provide a full impact assessment associated with pesticide use change with GM crops in any country. Therefore, despite the acknowledged weaknesses of the EIQ, it has been used in this paper because it is a superior indicator to only using amount of pesticide active ingredient applied.”*

## Results

### Areas, Varieties, Cost of Seed and Yields

The 735 farms in the survey farmed 636 ha of land and planted a total of 463 ha of corn (average farm area of 0.865 ha and average corn area of 0.63 ha). In addition, 44% of the farms also grew rice (where grown, an average area of 0.31 ha), which was typically the second most important farming activity. In relation to the corn production, just under three-quarters of the farms sold all or some of their production, with 41% consuming some or all of their production.

Varieties with the stacked GM traits were planted by 395 farmers on 251.8 ha, with the main varieties planted (by area) being DK 6919S, NK 7328 BT/GT, NK 4300 BT/GT, and DK 9955S which together accounted for over 90% of the total GM crop planted area. Conventional varieties were planted by 396 farmers, inclusive of 56 farmers who planted both GM and conventional varieties. The total area planted to conventional varieties was 211.2 ha, with the main varieties planted being NK 7328, HN 88 (waxy variety), CP 511, and NK 4300. Within the category of conventional varieties, nearest performing (conventional) ones to the varieties containing the GM traits were planted by 152 farmers on 90 ha. The nearest performing conventional varieties were the same varieties as those containing the GM traits, except with no GM traits added (NK66, NK 67, NK4300, NK7328, DK9955, DK6919, DK8868, DK6818, and CP 501).

**Figure 2.**
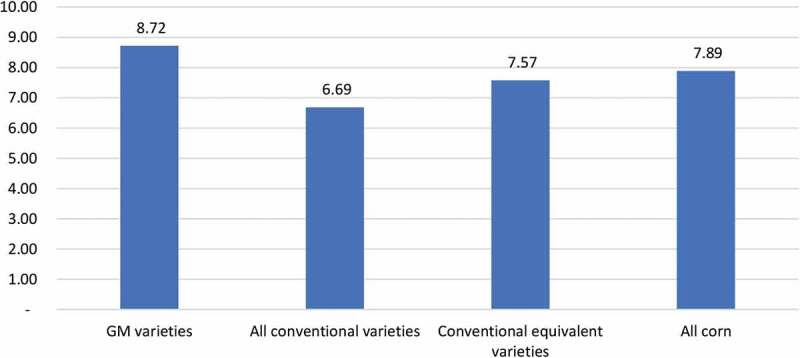
Average yield performance of different corn variety types (tonnes per ha)

Notes: 1. Yield figures are before de-husking and drying

The yield performance of the different varietal categories of corn grown is shown in Fig. 2.

The GM varieties, with an average yield of 8.72 tonnes per ha (5.45 tonnes per ha de-husked and dried), out-performed the average of all of the conventional varieties grown by +30.4% (+2.03 tonnes per ha or about 1.27 tonnes per ha de-husked and dried). In terms of a yield comparison between the nearest performing equivalent conventional varieties to the GM-traited varieties, the GM varieties outperformed the conventional equivalents by +15.2% (+1.15 tonnes per ha or 0.72 tonnes per ha de-husked and dried).

The main reasons for choice of conventional varieties were ease of selling the variety, familiarity with the variety and satisfaction with the yield level achieved (Table 2). In relation to the GM varieties, the most important reason cited for choice was high yield, followed by pest resistance, the value of requiring minimal care (in terms of needing to monitor for pest attack) and ease of weed control.

**Table 2.** Reasons for choice of variety

Reason	% of farmers in each category
*Conventional varieties (396 farmers)*	
High yield	19.2
Familiar with variety	17.7
Easy to sell	25.2
Soil suitability	5.3
Prefer this variety	4.1
Recommended by retailer or extension service	6.4
Good abiotic stress tolerance	4.9
High quality crop	3.0
*GM varieties (395 farmers)*	
High yield	30.5
Requires minimal care	14.0
Pest resistant	21.4
Ease of weed control	11.7
Overcomes weed resistance to paraquat	5.7
Recommended by extension service or farmer co-operative	9.4
Stable yield	4.0
High quality crop	6.3

For farmers using GM corn seed, the average cost paid for seed was US $130.8 per ha. For conventional “nearest equivalent corn varieties,” the average price paid for seed was US $97.38 per ha and the average price of seed for all conventional varieties was US $102.21 per ha. The (seed) premium paid by farmers for the GM traits was therefore equal to between US $28.59 per ha relative to nearest equivalent conventional varieties and US $33.42 per ha relative to the average of all conventional varieties planted.

#### Weed Control

Of the 395 farmers using GM corn seed, 82% used herbicides as the main form of weed control. Eighteen percent stated that they did not use herbicides but (continued) to use hand weeding as their main form of weed control. A small proportion of farmers (who used herbicides as their main form of weed control) also undertook some hand weeding (less than 20% of the total) or some mechanical weeding (less than 5% of the total). The main herbicides used were glyphosate, atrazine, and ametryn/atrazine, with glyphosate accounting for about half of the volume of herbicide active ingredient used, followed by atrazine and ametryn/atrazine which both accounted for about 15% each of the total amount of herbicide active ingredient used. The average cost of weed control when herbicides were used was US $45.72 per ha (comprising 48% for herbicides and 52% for application labor). Where supplemented with other forms of weed control, these costs were an average of US $12.86 per ha for hand weeding (an average of 10 hours per ha when used) and an average of US $13.49 per ha for mechanical weeding (3 hrs). Based on the proportion of the GM corn crop using each form of weed control, the weighted average cost of weed control was US $40.73 per ha (Table 3).

**Table 3.** Weighted average cost of weed control (US $ per ha)

	Average cost per ha	% of crop using each form of weed control	Weighted average cost of weed control
***GM corn***			
Herbicides	45.72	82	37.49
Hand weeding	12.86	21	2.70
Mechanical	13.49	4	0.54
***Total***			***40.73***
***Conventional corn***			
Herbicides	35.11	68	23.87
Hand weeding	117.60	58	68.21
Mechanical	33.62	4	1.34
***Total***			***93.42***

Note: values subject to rounding

Thirty-two percent of the farmers planting conventional varieties (127 farmers) indicated that they did not use herbicides for weed control on their conventional crops, relying on hand weeding only. Sixty-eight percent of the farms (269 farms) indicated that they made some use of herbicides. A small number (under 5% – less than 20 farms) supplemented their use of herbicides with some mechanical weeding. The main herbicides used on the conventional crop were atrazine, acetochlor, ametryn/atrazine, S metolachlor, and glufosinate, with atrazine accounting for about 55% of the volume of herbicide active ingredient used, followed by acetochlor and ametryn/atrazine (15% and 7% respectively of the total amount of herbicide active ingredient used).

The average cost of conventional corn weed control, when herbicides were used was US $35.11 per ha (comprising herbicides 46% and application labor 54%). Where supplemented with other forms of weed control, these costs were an average of US $117.6 per ha for hand weeding (an average of 126 hours per ha when used) and an average of US $33.62 per ha for mechanical weeding (17 hrs). Based on the proportion of the conventional corn crop using each form of weed control, the weighted average cost of weed control was US $93.42 per ha (Table 3).

#### Pest Control

Nineteen percent of GM corn farmers used insecticides. These farms were located mostly in Thai Nguyen (Northern Mid-land and Mountain region) and Ha Noi (Red River region). Where applied, the main insecticides used were emamectin benzoate, diazinon, permethrin, cypermethrin, and abamectin. The average expenditure on insecticide control, where used was US $55.65 per ha, of which 54% was for insecticide and 46% was for labor application.

Given that the GM seed technology provides control for the main corn pests, the circumstances relating to why these farmers were still making some use of insecticides were examined further. The main features were as follows:

- In 2019, FAW pest incidence was reported to be affecting between 35% and 75% of the corn area in most regions (source: personal communications from representatives of the seed industry), with the highest levels of incidence in the regions of North Mid-lands/Mountainous, Red River, and North Central. Experience of (controlling) this relatively new pest (in Vietnam) was limited and consequently, it is likely that some farmers did not realize that the GM corn seed provided control of this pest and therefore used insecticides for FAW control (notably the insecticide active ingredient emamectin benzoate). These farmers may also have made “insurance” applications of insecticide because they had no experience of how effective the seed was in controlling the pest. It is interesting to note, for example, that in the Son La district of the Northern Mid-land and Mountainous region, where interviews did not take place until late 2019 (when farmers had experienced performance of the GM seed in controlling the FAW pest in early 2019 crops), there was no recorded use of insecticides by GM farmers at all. Also, it is likely that some farmers experienced different levels of FAW control according to the traits present in the seed varieties used – where the traits provided effective control, no additional insecticide was used compared to using one additional insecticide application where the trait provided only partial suppression;
- Sixty percent of the GM farmers using insecticides were first time users of the seed. Therefore, it is possible that the lack of experience of using this technology and its efficacy levels in controlling pests may have contributed to some farmers making “insurance” applications of insecticide;
- Some of the insecticide use may have been used to control pests such as aphids, thrips, and the stored nut moth, which are not controlled by the GM traits

In addition, 60% of the GM corn farmers also said they devoted some time (an average 6 hours per ha) to scouting/crop walking for pest presence, at an average cost of US $13.58 per ha. Based on the proportion of the GM corn crop using insecticides and undertaking crop walking, the weighted average cost of pest control was US $18.72 per ha (Table 4).

**Table 4.** Weighted average cost of pest control (US $ per ha)

	Average cost per ha	% of crop using each form of pest control	Weighted average cost of pest control
***GM Corn***			
Insecticides	55.65	19	10.57
Crop walking	13.58	60	8.15
***Total***			**18.72**
***Conventional corn***			
Insecticides	35.15	72	25.31
Crop walking	38.84	80	31.07
**Total**			**56.38**

Note: values subject to rounding

In contrast, 72% of conventional corn farmers used insecticides for pest control (of all corn pests including those controlled by GM traits as well as aphids, thrips, and the stored nut moth). The average expenditure on insecticide control was US $35.15 per ha, of which 46% was for insecticide and 54% was for labor application. When applied, the main insecticides used were chlorfenapyr, chlorantraniliple, abamectin, emamectin benzoate, and spinetoram. Eighty percent of the conventional farmers also spent time (an average 17 hours per ha) scouting/crop walking for pest presence, at an average cost of US $38.84 per ha. Given the proportions of the conventional corn crop using insecticides and undertaking crop walking, the weighted average cost of pest control was US $56.38 per ha (Table 4).

#### Harvesting

The average amount of labor time spent on harvesting the GM crop was 92.5 hours per ha at a (weighted average) cost of US $124.90 per ha (Table 5). A large majority of the GM corn farms used adult family members (more than 80% of the total) to do harvesting, with just under 50% using some hired labor. The average amount of labor time spent on harvesting the conventional crop was 72 hours per ha at a (weighted average) cost of US $94.43 per ha. While a large majority of the conventional farms used adult family members (90% of the total) to do harvesting and 42% made some use of hired labor, the relative importance of family labor was greater than among the farmers growing GM corn.

**Table 5.** Corn harvesting costs and labor use (per ha)

Type of labor	Hours – where used	Cost per ha ($)	% of crop using each form of harvesting labor	Weighted average cost of harvest labor ($)
***GM crop***				
Adult family	40.9	55.21	81	44.72
Family – children	5.5	4.95	2	0.1
Hired	123.7	166.83	48	80.08
**Average all forms of labor**	**92.5**			**124.90**
***Conventional crop***				
Adult family	50	67.30	90	60.57
Family – children	17	16.36	2	0.33
Hired	64	79.84	42	33.53
**Average all forms of labor**	**72**			**94.43**

#### Farmers Views and Perceptions

The farmers who had grown GM corn were asked about any likes or benefits they perceived had occurred from using this type of corn. Improved pest and weed control were cited most frequently by 92% and 85% respectively of the farmers, with higher yields, cost saving, better grain quality, and higher income all registered as positive impacts by between 57% and 72% of the farmers. In addition, the 225 farmers (57% of the GM corn farmers) who actively stated that the use of GM corn had resulted in higher incomes also provided further information about the impact of having higher levels of income. The main impacts were having more money for on-farm investment and/or to spend on their families plus less need for family members to work on the farm.

In relation to perceived negative aspects of using GM corn, 158 farmers (40% of users) provided responses to this question. The main complaint, registered by 134 farms (34% of all GM farmers or 85% of this sub-set of responding farmers) was about the (high) price of seed. There were very few other complaints; less than 4% of farmers (15 farmers) said they perceived there might be “negative health” issues associated with use of the technology (based on information they had read in the media) and 2% (8 farmers) said they were disappointed with the yield performance.

Lastly, the likelihood of farmers choosing to grow GM corn again in the next season, only two farms (0.5% of the total) said they would not plant GM corn next season.

Conventional corn growers were asked why they had not tried using GM corn. One hundred and sixteen conventional corn growers offered responses to this question with the main reason cited (by 57% of the respondents: 66 farmers) being the (high) price of the seed. In addition, 29% of these farmers (34 farmers) indicated that they perceived that the benefit would be less than the expected higher cost of the seed. Lastly, 10 farmers (9% of this group) were growing specialty varieties of waxy corn and said if they switched to using GM seed (the GM traits are not available in any waxy corn varieties), they would expect to see income levels fall because any agronomic gains would be offset by the higher cost of seed and lower value of the (non-waxy) corn.

### Overall Yield, Cost of Production and Income Impacts

The main impacts of using GM corn have been (Table 6):

- The GM varieties out-performed conventional varieties in terms of yield by +30.4% (+2.03 tonnes per ha or about 1.27 tonnes per ha de-husked and dried). In relation to a yield comparison between the nearest performing equivalent conventional varieties to the GM-traited varieties, the GM varieties outperformed the conventional equivalents by +15.2% (+1.15 tonnes per ha or 0.72 tonnes per ha de-husked and dried). In revenue terms this amounted to an increase of between US $169.20 per ha and US $298.45 per ha;
- There have been changes in the type and nature of herbicides applied (more use of the broad-spectrum herbicide glyphosate and less use of pre-emergent herbicides like atrazine and acetochlor). There has also been a reduction in the use of hand weeding. While average expenditure on herbicides and their application has increased, this has been more than offset by savings from less use of hand weeding. Overall, the average cost of weed control has fallen by US $52.69 per ha;
- There have been reductions in the use of insecticides for the control of pests, with the control of the main lepidopteran pests now provided via the seed, leaving residual use of insecticides for the control of pests not controlled by the GM traits. These savings amounted to US $14.75 per ha. In addition, farmers using GM varieties spent less time on crop walking/scouting checking pest levels. This resulted in an additional cost saving equal to US $22.93 per ha;
- The higher yields derived from GM corn have required additional use of labor for harvesting. This extra cost has been an average of nearly US $30.46 per ha;
- The cost of seed has increased, with the seed premium for GM corn seed being an average of between US $28.60 per ha (average to all conventional varieties used) and US $33.43 per ha (average to equivalent performing varieties to the GM varieties);
- Overall, the net impact on farm income associated with using GM corn has been an increase in the average level of farm income of between US $196 per ha (relative to equivalent conventional varieties) and US $330 per ha (average of all conventional varieties);
- It is interesting to note that 18% of the farmers using GM corn did not use herbicides for weed control even though the seed contained tolerance to the herbicide glyphosate. In effect, these farmers used the seed specifically for its pest control capability rather than its potential for improving weed control. While these farms will have foregone potential weed control cost savings (as derived by other users of the technology), the savings associated with lower pest control costs and higher yields were still significant;
- An important production impact highlighted by 60% of the GM corn users was improvements in the quality of the grain. While the farmers who highlighted this impact did not provide additional information about the nature of these quality improvements, it is likely, based on the findings of analysis of the impact of using GM corn in other countries (e.g., Folcher et al^8^ Bakan et al^9^ Munkvold et al^10^ Wu^11^) that this relates to reduced levels of aflatoxins and fumonisins in the GM crop grain compared to conventional corn. This can result in reduced levels of wastage and/or rejection by purchasers (of the grain), notably in the food using sector.

**Table 6.** Summary of farm-level income impact of using GM corn (US $ per ha)

	GM	Conventional: all	Conventional: nearest equivalent varieties to GM
*Crop yield (tonnes per ha)*	8.72	6.69	7.57
Crop yield de-husked/dried (tonnes per ha)	5.45	4.18	4.73
Price 2019	235	235	235
Revenue	1,280.75	982.30	1,111.55
**Yield difference (tonnes per ha)**		**1.27**	**0.72**
**Revenue change**		**+298.45**	**+169.20**
*Cost of seed*	130.81	102.21	97.38
**Seed premium**		**− 28.60**	**− 33.43**
*Weed control costs: weighted average*			
Herbicides	37.49	23.87	23.87
Hand weeding	2.70	68.21	68.21
Mechanical	0.54	1.34	1.34
total cost	40.73	93.42	93.42
**Change in weed control costs**		**52.69**	**52.69**
*Pest control costs*			
Insecticides	10.57	25.31	25.31
**Change in insecticide costs**		**14.74**	**14.74**
Crop walking/scouting	8.15	31.08	31.08
**Change in crop walking/scouting costs**		**22.93**	**22.93**
*Harvesting costs*	124.89	94.43	94.43
**Change in harvesting costs**		**− 30.46**	**− 30.46**
Total change in costs of production		31.30	26.47
**Total change in income in US $ terms**		**+329.75**	**+195.67**

Exchange rate US $1 = 22,244 (2019 average)

Note: – ve sign = increase in costs

Values subject to rounding

Examining the cost farmers pay for accessing the GM seed technology, the average additional cost of seed (seed premium) relative to conventional seed, over the period of adoption was between US $28.60 per ha and US $33.43 per ha. These cost of technology values are equal to between 8% and 15% of the total (gross) technology gains (before deduction of the additional cost of the technology payable to the seed supply chain – the cost of the technology accrues to the seed supply chain including sellers of seed to farmers, seed multipliers, plant breeders, distributors, and the GM technology providers). In terms of investment, this means that for each extra dollar invested in GM corn seed (relative to the cost of conventional seed), farmers gained an average of between US $6.84 and US $12.55 in extra income.

The main changes in labor use associated with the adoption of GM corn were:

- A reduction in the total amount of labor used per ha of about 71 hours per ha (−8.9 days). This derives from less use of labor for weeding (largely hand weeding), application of insecticides and crop walking/scouting. Some of these labor reductions were offset by an increase in the labor requirement for harvesting;
- As the majority of the labor used on farms has been family labor, the labor requirement changes have mostly impacted on this category of labor. For hired labor, the adoption of GM corn has resulted in a small net increase in labor requirement because of the increased requirement for harvesting. While the requirement for hired labor to undertake weed control (and to a lesser extent pest control) has decreased, this has been more than offset by an increase in hired labor use for harvesting. Overall, the hired labor requirement has increased by about 10 hours per ha (1.25 days per ha);
- The overall reduction in labor use on GM corn growing farms, which has mostly impacted on the use of family labor was acknowledged by many of these farmers as a positive aspect of change because it had freed up more time for farmers and their family to spend on other income-generating activities and leisure.

### Environmental Impact Associated with Herbicide Use for Weed Control

GM farmers increased their use of herbicides as the primary form of weed control relative to the weed control practices of conventional corn producers. However, the profile and volume of the herbicides used have changed, with the average amount of herbicide active ingredient used and its associated environmental impact, as measured by the EIQ indicator being lower for GM corn adopters than conventional corn growers. The average amount of herbicide active ingredient applied on the 82% of the GM crop area that used herbicides for weed control was 2.08 kg/ai per ha, compared to 2.88 kg/ai per ha on the 68% of the conventional corn crop area that used herbicides for weed control. When this herbicide usage is averaged across the respective total areas planted to GM and conventional corn, the average amount of herbicide applied to the GM corn crop was 26% lower (1.66 kg per ha) than the average value for the conventional corn area (2.26 kg/ai per ha).

In terms of the associated environmental impact of the herbicide use, as measured by the EIQ indicator, the average EIQ value or load per ha of herbicide active ingredient applied on the 82% of the GM crop area that used herbicides, was 37.95 per ha, compared to 59.74 per ha on the 68% of the conventional corn crop area that used herbicides. When this herbicide usage is averaged across the respective total areas planted to GM and conventional corn, the average EIQ load per ha was also lower by 36% (30.26 per ha) than the average value applicable to the conventional corn area (46.95 per ha).

### Environmental Impact Associated with Insecticide Use for Pest Control

The adoption of GM corn has led to changes in pest control practices, with GM farmers applying insecticide to a significantly lower crop area and, where used, in smaller amounts.

On the 19% of the GM crop area that used insecticides, the average amount of insecticide applied was 0.42 kg/ai per ha, compared to an average of 0.5 kg/ai per ha on the 72% of the conventional corn crop area that used insecticides. When this insecticide usage is averaged across the respective total areas planted to GM and conventional corn, the average amount of insecticide applied to the GM corn crop was significantly lower by 78% (0.08 kg/ai per ha) than the average value for the conventional corn area (0.36 kg/ai per ha).

In terms of the associated environmental impact of the insecticide use, as measured by the EIQ indicator, the average insecticide EIQ load per ha value on the 19% of the GM crop area that used insecticides was 17.26 per ha, compared to an average of 19.65 per ha on the 72% of the conventional corn crop area that used insecticides. When this insecticide usage is averaged across the respective total areas planted to GM and conventional corn, the average EIQ per ha was significantly lower by 77% (3.23 per ha) than the average value for conventional corn (14.06 per ha).

### Views, Experiences and Perceptions of GM Corn

The vast majority of farmers who had grown GM corn expressed high levels of satisfaction with the technology and no negative impacts were identified in the farm survey, other than a number of farmers (less than 10% of users) indicating that perceived that the additional cost of the seed was too high. Despite this, 99.5% of adopters stated that they would be using the technology in the 2020 crop year. The high levels of satisfaction were linked to the benefits associated with adoption, of improved levels of pest and weed control, higher yields, higher incomes, and better grain quality. In addition, the higher levels of income had resulted in farmers having more money for on-farm investment and household expenditure. Also, there has been reduced need for family members to work on farms, allowing more time for leisure activities.

In relation to conventional corn growers the main reasons cited for not trying the new technology was the (perceived) high price of the seed relative to conventional seed and/or the view that the benefit (of adoption) would be less than the extra cost of the seed. In addition, growers of specialty varieties of waxy corn said if they switched to using GM seed (the GM traits are not available in any waxy corn varieties), they would expect to see income levels fall because any agronomic gains would be offset by the higher cost of seed and lower value of the non-specialty corn.

#### Aggregated Impacts

The aggregated farm-level income impact of using GM corn, based on the survey findings and applied to the national level of GM corn seed adoption in 2019 (92,000 ha or 10.2% of the national crop) was a net income gain of between US $17.95 million (based on the yield gain relative to the nearest equivalent varieties to the GM varieties) and US $30.38 million (based on the yield gain relative to all conventional varieties). The vast majority of the income gains (more than 90%) came from yield gains, with some small additional benefits associated with lower net costs of production.

If these farm income gains are applied to the cumulative GM crop area since the technology was first used in 2015 (a total of 224,500 ha 2015–2019), the total farm income gain has been between US $43.8 million (based on the yield gains relative to the nearest equivalent conventional varieties) and US $74.1 million (based on yield gains relative to all conventional varieties).

Based on the yield gains referred earlier, the GM corn technology added between 66,000 tonnes and 117,000 tonnes of corn in 2019 and cumulatively since 2015 has added between 157,900 tonnes (based on the yield gains relative to the nearest equivalent conventional varieties) and 315,400 tonnes (based on yield gains relative all conventional varieties).

At the national level, the aggregate labor impact in 2019 is small and equal to a net reduction in labor use of about 6.52 million hours or 815,000 days (or 3,400 full-time equivalents – FTEs). Most of this reduction in labor use has affected family labor. In terms of hired farm labor, the impact has been a net increase of about 0.92 million hours (115,000 days), or 480 FTEs, primarily for additional crop harvesting work.

In relation to weed control changes, the use of GM corn (on the equivalent of 10.2% of the total corn crop) resulted in a reduction in the amount of herbicide active ingredient used on the whole crop of 2.7% (−55,220 kg) and a net reduction in the associated EIQ value of 3.6% (Table 7). Cumulatively, since 2015, there has been a net decrease in the amount of herbicide active ingredient used of 134,760 kg (−1.2% of total crop use over this period) and a net reduction in the environmental load associated with herbicide use, as measured by the EIQ indicator of 1.5%.

**Table 7.** Insecticide and herbicide use changes with GM corn: 2019 and cumulative 2015–2019

	Area of trait (ha)	Average reduction in ai use (kg per ha)	Average reduction in field EIQ per ha	Aggregate change in ai use (‘000 kg)	Aggregate change in field EIQ per ha units (‘000s)
Insecticides 2019	92,000	0.28	10.83	25,440	996
Herbicides 2019	92,000	0.60	16.69	55,220	1,536
Insecticides cumulative	224,500	0.28	10.83	62,075	2,431
Herbicides cumulative	224,500	0.60	16.69	134,760	3,747

In terms of the pest control, the use of GM corn has led to a reduction in the amount of insecticide active ingredient used on the whole crop of 25,440 kg (−7.95% in terms of all insecticide used on the crop) and a net reduction in the associated EIQ value of 7.87%. Cumulatively, since 2015, there has been a net decrease in the amount of insecticide active ingredient used of 62,075 kg (−3.4% of total crop use over this period) and a net reduction in the environmental load associated with insecticide use, as measured by the EIQ indicator of 3.3%.

## Discussion and Conclusion

The evidence identified in the farm survey shows that GM corn adoption in Vietnam has delivered significant socio-economic benefits to the farms that have used the technology. There have also been wider (to society) environmental benefits associated with changes to weed and pest control practices that have reduced the environmental load associated with pesticide use on corn.

The yield gains, at more than +15% (and up to +30%) are at the higher end of the range of performance of similar GM seed technology in other developing countries^3^ (e.g., Philippines +23.2%,^3,12–15^ South Africa +11.1%,^3,16–19^ Colombia +17.4%,^20,21^ Honduras +23.9%.^3,22,23^) They are also higher than the expected +5% to +12% which were based on historic estimates of yield losses associated with ACB pest damage and weed competition in the conventional crop plus 2015 field trials of the stacked maize in Vietnam.^4^

Largely due to the high yield benefits derived, the farm income gains derived by farmers (+US $196 per ha to +US $330 per ha) are also at the higher end of the range of performance for similar technology in other adopting countries (e.g., +US $101 per ha in South Africa, +US $294 per ha in Colombia, +US $131 per ha in Philippines).^3^

It is also worthy of note that with the incidence of the relatively new corn pest, the FAW, in Vietnam in 2019, the positive yield impacts identified in the survey may have begun to include a contribution from the effective control to this pest in the regions where this pest has become established, while conventional corn growers have suffered additional yield losses and incurred additional costs for extra insecticide applications.

The average return on investment (relative to every extra US $1 cost of GM seed) of between US $6.84 and US $12.55 is also one of the highest rates of return earned by any GM crop-adopting farmers in the world. It is broadly equivalent to the level of returns earned by GM cotton farmers in India and China and is significantly higher than the returns of GM corn farmers, for example, in the Philippines have achieved (of about US $2.63).^3^

The small net decrease in employment requirements on-farm, is also consistent with the labor impacts identified in other countries that have used the same GM seed technology in corn and cotton (e.g., corn in South Africa,^17^ cotton in India^24^).

Evidence of possible negative impacts associated with adoption of this corn seed technology in other countries such as incidence of pests becoming resistant to the GM technology (e.g., Tabashnik and Carrière^25^) or to the development of weeds becoming resistant to glyphosate (as discussed in the introduction) were not found in this study. Whilst the authors acknowledge that incidence of pest and weed resistance issues could arise in the future, as indicated in the introduction, the scope for pest resistance developing has been reduced through IRM strategies provided by the technology providers. In addition, the risk of weeds developing resistance to glyphosate has been reduced through extension advice and training provided by the GM seed suppliers to farmers before they use the seed, for example, that advises that if glyphosate is being used for “over the top of crop” weed control with GM corn, that farmers should ensure that different forms of weed control (including, e.g., mechanical or hand weeding) or other herbicides (with different modes of action) are also used, especially at the soil preparation and early, pre-emergence phase of crop growth (source: personal communication from representatives of the seed industry).
