# Correction

**DOI:** 10.1080/21645698.2021.1843218

**Published:** 2020-11-09

**Article title**: The impact of using genetically modified (GM) corn/maize in Vietnam: Results of the first farm-level survey

**Authors**: Graham Brookes and Tran Xuan Dinh

**Journal**: *GM Crops & Food*

**DOI**: http://dx.doi.org/10.1080/21645698.2020.1816800

When this article was published online, some minor factual inaccuracies in two places in the text were noticed. The article has now been corrected and the corrections are also included below.

## Introduction – in paragraph 3

The ‘stacked-traited’ seed technology is available to farmers in a limited number of yellow corn hybrid varieties that have been approved for use in Vietnam, notably NK66 BT/GT, NK 67 BT/GT, NK4300 BT/GT, NK7328 BT/GT, DK9955S, DK6919S, DK8868S, DK6818S and CP 501S. The technology is, however, not available in some of the latest developed hybrid varieties, is not available in specialty varieties (eg, waxy corn used in the starch manufacturing sector), in sweetcorn or in open pollinated varieties. In order to minimize the incidence of pests (especially those such as Ostrinia furnacalis which are polyphagous and feed on more than one crop species) becoming resistance to the IR technology, Insect Resistance Management (IRM) strategies are adopted. These are implemented through the technology provider. An appropriate IRM strategy includes the use of, what are referred to as, unstructured or structured refuges. An unstructured refuge is where other forms of non IR plants or wild hosts of the targeted pests are planted, whilst a structured refuge generally refers to a refuge planted as a separate block or field from the IR protected crop. Structured refuges can, for example, be provided by supplying a small package of refuge seed, along with the larger amount of IR-traited seed or a seed blend of the two types of seed. Along with a refuge-based IRM, a pyramid of more than one than one Bt protein can play a role in the IRM strategy.

## Discussion – last paragraph

Evidence of possible negative impacts associated with adoption of this corn seed technology in other countries such as incidence of pests becoming resistant to the GM technology (e.g., Tabashnik and Carrière^25^) or to the development of weeds becoming resistant to glyphosate (as discussed in the introduction) were not found in this study. Whilst the authors acknowledge that incidence of pest and weed resistance issues could arise in the future, as indicated in the introduction, the scope for pest resistance developing has been reduced through IRM strategies provided by the technology providers. In addition, the risk of weeds developing resistance to glyphosate has been reduced through extension advice and training provided by the GM seed suppliers to farmers before they use the seed, for example, that advises that if glyphosate is being used for “over the top of crop” weed control with GM corn, that farmers should ensure that different forms of weed control (including, e.g., mechanical or hand weeding) or other herbicides (with different modes of action) are also used, especially at the soil preparation and early, pre-emergence phase of crop growth (source: personal communication from representatives of the seed industry).

